# Convolutional Neural Network-Based Human Movement Recognition Algorithm in Sports Analysis

**DOI:** 10.3389/fpsyg.2021.663359

**Published:** 2021-06-25

**Authors:** Jiatian Liu

**Affiliations:** College of Strength and Conditioning, Beijing Sport University, Beijing, China

**Keywords:** human action recognition, convolutional neural network, image recognition, sports analysis, sports psychology

## Abstract

In order to analyse the sports psychology of athletes and to identify the psychology of athletes in their movements, a human action recognition (HAR) algorithm has been designed in this study. First, a HAR model is established based on the convolutional neural network (CNN) to classify the current action state by analysing the action information of a task in the collected videos. Secondly, the psychology of basketball players displaying fake actions during the offensive and defensive process is investigated by combining with related sports psychological theories. Then, the psychology of athletes is also analysed through the collected videos, so as to predict the next response action of the athletes. Experimental results show that the combination of grayscale and red-green-blue (RGB) images can reduce the image loss and effectively improve the recognition accuracy of the model. The optimised convolutional three-dimensional network (C3D) HAR model designed in this study has a recognition accuracy of 80% with an image loss of 5.6. Besides, the time complexity is reduced by 33%. Therefore, the proposed optimised C3D can recognise effectively human actions, and the results of this study can provide a reference for the investigation of the image recognition of human action in sports.

## Introduction

The basketball player with the ball applies footstep movement and dribbling skills to break through and quickly pass the defence of the opposing player. In the process of ball holding, players can effectively promote the chances of breaking through the defence by taking advantage of their own speed and reasonable use of fake actions (Zhou et al., 2016). Fake action refers to how athletes use the illusion of their own movement techniques to confuse the vision and feeling of the opposing players so that the opposing athletes make wrong judgments or lose their own body balance. What’s more, it can conceal their own movement intention, realise their own real purpose of sports, and gain the advantage of time and space on the court (Qi et al., 2019). Therefore, the reasonable application of fake action can specifically reflect the psychology of athletes in sports. The artificial intelligence algorithm based on human–computer interaction is adopted to recognise the actions of basketball players in the game. In combination with the basketball movement rules and the relevant theories of psychology and physiology, analysis and investigation have been conducted for the fake actions of basketball players to obtain qualitative and quantitative results (Du et al., 2020).

Chen et al. (2018) proposed two deep convolutional neural networks (DCNNs) based on multi-static radar micro-Doppler signals for human recognition and gait classification. The directional diversity provided by polymorphic radar was applied to improve the classification accuracy. Firstly, a single static DCNN was proposed based on voting. Then, it was trained on each receiving node and the results were fused by binary voting. The fusion steps were incorporated into the network structure, and the data were validated by the multi-static DCNN. The experimental results indicated that its classification accuracy was more than 99%. Jing et al. (2020) put forward a recurrent neural network for skeleton-joint collaborative recognition to capture motion sequences of skeleton-joint feature images, so as to obtain spatial consistency between joints and the time evolution process between bones. The next motion was predicted and simulated by dynamically learning the motion information of the skeleton-joint feature map. In addition, Hu et al. (2020) have presented a network structure combined normalisation algorithm with convolutional neural network (CNN) model to extract features in human actions. Firstly, there was a normalisation for the training sample of network input. Secondly, red-green-blue (RGB) and stacked optical flow have been considered as spatial and temporal inputs of CNN, respectively. Finally, the results have been fused, revealing that the recognition accuracy of the network structure is 93.42% to the video.

The objective of this study is to explore the psychology of basketball players when displaying fake actions in the attacking and defending process. DNN is employed in this study to establish the human action recognition (HAR) model to identify the actions of players in sports. According to the recognition and judgment of the current action of the athlete, the next action is predicted in combination with the relevant sports psychology theories, so that the defender could effectively block the ball-holding breakthrough of an attacker with a cross-step. First of all, there is an introduction of CNN, and the optimised convolutional three-dimensional network (C3D) is designed to identify human actions. Then, the psychology of the attacker is analysed on the basis of the related sports psychology theories. Finally, the network parameters of the HAR model are selected, so that its recognition effect is tested.

## Materials and Methods

### HAR Algorithm Based on CNN

Human action recognition has been achieved through its decomposition, and human action can be divided into three categories (Wang et al., 2018d). The first one is the basic movement unit, such as raising hands, stepping, and turning around. The second one is the human movement, including the overall movements formed by basic movements (raising hands and turning around) during stepping. The third one is the human activity that refers to the actions performed by the person to complete the entire event, such as dancing, running, and other movement processes composed of a series of actions. Therefore, video data or image sequences are used for the identification of actions of the human body based on the breakdown of human actions. Besides, the video capture device is adopted to collect the movements of the human body (Wang et al., 2018c). Then, the collected video data are analysed and processed to decompose the human actions in the video sequence, thereby extracting the action features (Chen and Shen, 2019; Gurbuz and Amin, 2019). Thus, the actions can be judged and classified through the human action characteristics.

Convolutional neural network is an image recognition algorithm based on the visual perception process of biology. Compared with a traditional neural network, this algorithm adopts a convolution computation. The neurons between the convolutional layers of CNN are only connected with a few neurons between the adjacent layers, and the pooling and convolutional layers can respond to the translation invariance of the input features, effectively identifying the similar features of images (Bu, 2020; Gao et al., 2020). Moreover, CNN is composed of a convolutional layer for convolution operation, a pooling layer for feature screening, and a fully connected layer for feature fusion (Chen, 2019). Each layer of the convolutional layer has a large number of neurons. When neurons are trained, each neuron has a weight value. The convolutional layer applies a nonlinear function to map the neurons, and the pooling layer will process them according to the corresponding weight values. Then, they are passed to the next layer (Zhang et al., 2019) so that the results are output and classified in the fully connected layer.

The convolutional layer is the convolution operation of the input original image and video frame to obtain the underlying features contained in the image. During the convolution process, the convolution kernel is applied to extract the features of the input image. One convolution kernel can extract one image feature, and each convolution kernel has a corresponding weight coefficient and offset (Zhao, 2016; Wang et al., 2018e). In the first-level convolution process, the convolution kernel scans the input image based on the step size, and the obtained data are convolved to get the low-level features. Then, the nonlinear function is adopted for result processing to gain the convolution graph of the first layer network. In the middle layer of the convolutional layer, the output results of the previous layer are trained and learned based on the weight parameters and offsets within the layer. Furthermore, the images are convolved with different receptive fields of the same convolution kernel, reducing the parameter settings and complexity of the network (Vu and Kim, 2018; Xia et al., 2020). In the middle layer of the convolutional network, the high-level features of the image can be obtained through multiple feature extraction (Kamel et al., 2018).

The pooling layer can compress the feature images extracted by the convolutional layer and select regions in the feature image. Besides, the data features are selected based on correlation, so as to extract new data as new features of the image (Nunez et al., 2018). The pooling operation can reduce the data dimension and resolution of the image and the influence of data noise on the network. Pooling includes two methods, namely maximum pooling and average pooling. Maximum pooling is to extract the maximum value of the image through a sliding window. After extraction, the features of the convolutional layer are pooled maximally to identify the feature value and narrow the size of image (Saeed et al., 2019; Yang et al., 2019). Average pooling is to extract the average value of the image through a sliding window. In the final convolution kernel pooling operation, the feature images are expanded and connected into vectors. Finally, the results are input to the fully connected network (Dehzangi et al., 2017). The feature images obtained by the convolution kernel pooling can be combined with the fully connected layer. Considering that all the feature relationships need image stitching, the 3D structure of the feature image is removed, and the vector to the next layer is passed through the excitation function (Zheng and Ke, 2020; Zheng and Liu, 2020).

Since the human action is a continuous process (an action will last dozens of frames in the video), the 3D CNN can extract the temporal and spatial characteristics and timing information of the motion information in the video. 3D CNN is to expand the time dimension, so that continuous image frames are operated convolutionally. When the images of a video are processed, the video is divided into video frames and input into the CNN in accord with the set number of frames. The collection of human actions is affected by environmental light and field of view, and so the recognition of human actions in the video needs to consider the spatial characteristics and sequence of human actions between video frames (Li et al., 2017). The graph convolutional network can identify irregular data in human actions and is used for bone modelling to fuse different nodes in the image, so as to obtain an image with structural information. For example, the application of Poincaré geometric shapes has been studied in the literature (Peng et al., 2020) to graph convolutional networks, thus establishing the spatiotemporal graph convolutional network to better model the potential anatomy of structural data. In C3D, a video frame cube is built by constructing a few consecutive frames of images, and there is a convolution inside the cube. The convolution operation for the timing sequence is achieved by the three consecutive video frames through a 3D convolution kernel, which is executed by applying a variety of convolution kernels, thereby improving the ability of network feature extraction (Wang et al., 2018a).

**FIGURE 1:**
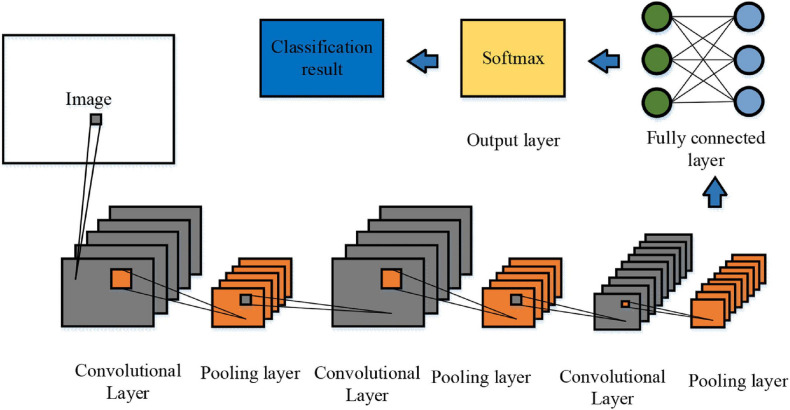
Structure of the optimised C3D.

In order to enhance the network’s ability to learn image features, two 1 × 3 and 3 × 1 convolution kernels are used for convolution operations on images so that more image information can be obtained under the same convolution kernel receptive field. Besides, the number of filters and activation functions is added, the nonlinear learning ability of the neural network is enhanced, and the occurrence of over-fitting is prevented. Finally, the obtained data is integrated at the fully connected layer (Hou et al., 2016). The structure of the optimised C3D is shown in Figure 1, including three convolutional layers, three pooling layers, two fully connected layers, and one softmax layer. The image was randomly cropped during the training process, and the initial learning rate was 0.003. When C3D is trained, the parameters of the pre-trained model are fine-tuned to improve its performance.

The computer has a greater pressure to read the image data because the images of video frames are composed of three primary colours. The collected images are grayed in order to increase the speed of the computer, thus ignoring the light, shadow, and colour changes of human actions. The characteristic information contained in the image is lost, which is called image loss. During the training process, the relevant parameters of the model need to be set. For this reason, the gray-scale processed video frames are input into C3D. Due to the consistency of the gray-scale image data, the gray-scale images are selected to determine the initial network parameters (Wang et al., 2018b; Zhang and Cao, 2018; Shen et al., 2019).

The recognition rate is selected as the evaluation index of the model, which can be calculated as follows:

(1) $$a\,c\,c\,u\,r\,a\,c\,y=\frac{T\,P+T\,N}{T\,P+T\,N+F\,P+F\,N}$$

In the Eq. 1, *TP* stands for the number of positive samples that are correctly classified; *TN* expresses the number of negative samples that are correctly classified; *FP* means the number of positive samples that are incorrectly classified; *FN* represents the number of negative samples that are incorrectly classified.

### Psychological Analysis of Fake Actions in Ball-Holding Players With Cross-Step

The reaction time is the time required for the human body to respond to stimuli, which is regarded as an index to measure the excitement of neuromuscular tissue and the speed of human reaction. In basketball, the reaction time can effectively evaluate the mental activity of the athlete, which represents the time distance from the stimulation to the reaction of the athlete. What’s more, reaction time can be divided into simple reaction time and complex reaction time. The simple reaction time is the time for the response action when there is a single conditional stimulus. As for the complex reaction time, it is the time distance for an athlete under complex reaction conditions to make a necessary response action when receiving multiple stimuli. The reaction time includes three conditions. The first one is the time when the receptor is stimulated and passed through the neuron. The second one is the time for the nerve impulse to be transmitted from the neuron to the cerebral cortex and from the motor centre to the effector. The third one is the time when the effector receives the stimulus to cause movement. Above all, the reaction time is an important psychological feature of the human, affecting the athletic ability (Mohr et al., 2016; Schweizer et al., 2020).

Selective attention refers to the situation in which a person only focuses on some stimuli or aspects and ignores others in the face of many stimuli from the outside world, and its essence is the aggregation and concentration of consciousness. Selective attention in sports means the state in which the athletes’ mental activities make corresponding selective response actions through consciousness adjustment to various sports technology-related stimuli. The decision is made primarily through the stimulation of visual information for all possible stimuli related to the completion of the action to achieve the best performance. Visual attention plays a decisive role in energy competition and is a critical psychological guarantee for athletes to achieve good results (Giovanini et al., 2020). As for basketball players, they face a lot of information and need to make effective response choices in a very short time, so the choice of relevant information becomes the basis of winning.

Ball-holding breakthrough is a very strong offensive technology, and the successful ball-holding breakthrough can increase the chances of shooting and passing in the basketball game, so as to disrupt the opponent’s defensive rhythm. Basketball sports techniques include linear motion and compound motion of the human body or the ball. From the perspective of body balance, there are two kinds of balances, that is, static balance and steady-state balance (Latinjak et al., 2019). Static balance means that the human body’s action remains static relative to its spatial position for a period of time. Besides, steady-state balance indicates that athletes adjust their postures and actions to combine multiple technical movements in order to achieve the effect of sports techniques. Moreover, they maintain the dynamic balance of the body in the whole process. Therefore, the ability to effectively judge the footstep of opposing players in the game is the key to winning (Stambulova and Wylleman, 2019).

In the process of ball-holding breakthrough, fake action reflects the wisdom of basketball players. Most importantly, there is a small gap in the speed, strength, and physical ability of athletes with the progress of science and technology and the improvement of sports training theory. Therefore, the reasonable application of fake actions in competitions becomes a crucial influencing factor for the decisive games (Mateus et al., 2020). In the basketball game, solid and collective defence increases the limitation of offense space. Through the effective control of individual action and the application of attack tactics, the sharp-witted response of the attacker is cooperated with the fake actions to cause visual disturbance to the defender and expand the psychological refractory time, thereby verifying the defensive reaction time of the opponent to increase the breakthrough defensive success rate (Tejero et al., 2017).

**FIGURE 2:**
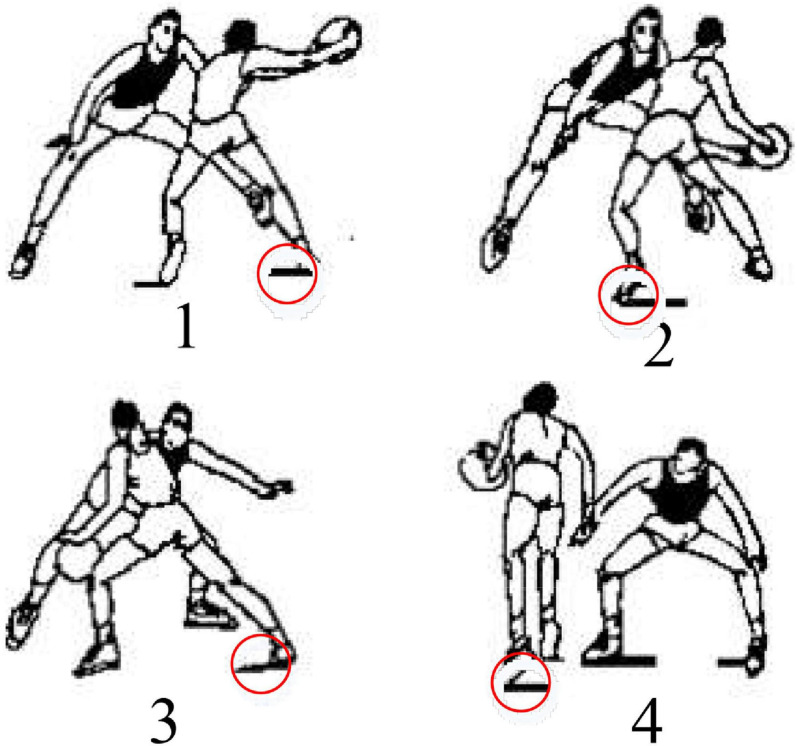
Analysis on the movement of right false shaking to the left.

Different fake actions have different influences on the defender. In the face of the attacker’s cross-step breakthrough with the ball, the final response action will be affected by the judgment of left false passing to the right, right false passing to the left, left false shaking to the right, and right false shaking to the left. The faster the selective response of the defender, the shorter the reaction time of the defence (Hobson et al., 2018; Kruger et al., 2018). In the process of breaking through the opponent’s defence, the defender will have 2 sides (one side with the ball and the other without the ball), so the attacker can take advantage of that to better attack. There are differences in the footsteps of basketball players, so that the reaction speed of attacker is related to the selective reaction time and moving footstep (Atkins et al., 2020; Hannington et al., 2020). There is an investigation of the human actions of the cross-step ball-holding player when displaying fake actions to face with the opposing defence in the breakthrough. The defence and offense process of 1 vs. 1 basketball game is analysed in terms of the application of psychology, physiology, and basketball movement rule. The fake actions of the defender are also analysed in the face of the attacker, including the left false passing to the right, right false passing to the left, left false shaking to the right, and right false shaking to the left (Figure 2), thereby gaining an effective judgment. When training the neural network, the training set and parameters can be adjusted to judge and predict the state of the athlete’s foot force, so as to effectively obtain the action intention and identify the fake action intention. The red circle of Figure 2 indicates the change of the athlete’s foot force state when he is performing a movement of right false shaking to the left.

In this study, six members of a men’s basketball team in City A were selected as the research objects, with a professional training period of over 3 years. Besides, they received pre-experimental training. In the experiment, high-speed cameras were applied to film the 1 vs. 1 attack and defence process of the athletes, and a reaction time measuring instrument was employed to test the reaction time of the athletes’ fake actions. We posited that players should display a variety of fake actions, such as breakthrough bypassing to the right and false passing to the left, a breakthrough by false passing to the left and shaking the right, and dribbling the ball in the opposite direction. In the 1 vs. 1 offensive and defensive process, both parties try their best to give play to their own technical characteristics. During the offensive process, the attacker makes a fake breakthrough that is recorded by the camera. The completion of fake actions should be in line with the regulations. The athletes complete all the fake actions, indicating that the experiment is finished. Then, the team members switch positions, and each of them should complete the experiment twice. Finally, the designed HAR model is applied to analyse these videos to obtain the reaction time data of the players’ defensive actions. As a result, the shooting and data processing of the experiment meets the needs of scientific research.

## Results and Discussion

**FIGURE 3:**
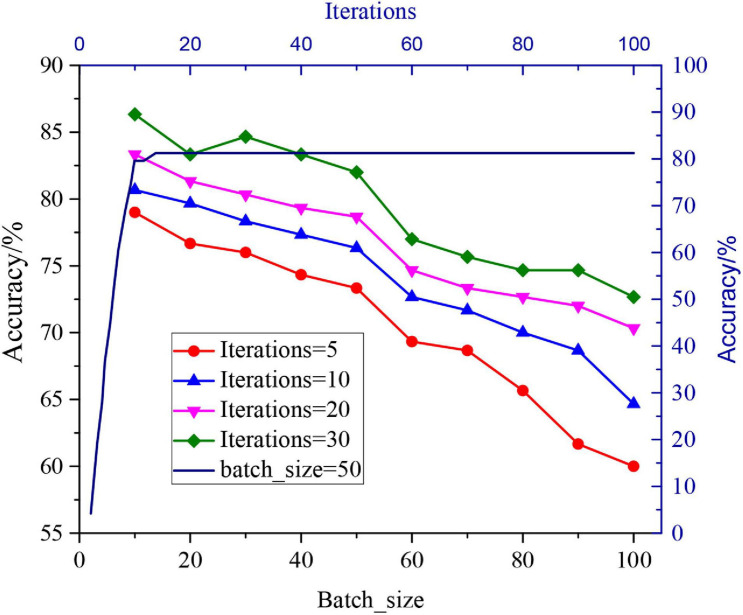
Influence of different batch sample numbers and iteration times on the recognition effect.

In order to train the designed human recognition algorithm, the UCF101 database and HMDB51 database are used as the training database of human behaviours on Matlab R2014a. The designed algorithm is adopted to extract the image features, and then, the fusion result is classified by the classifier. In the network training, 5, 10, 20, and 30 iterations are adopted to test the batch sample number of 1-100, in order to determine the batch sample number of the samples, respectively. The results are shown in Figure 3.

Figure 3 reveals that the number of batch samples is negatively correlated with the recognition rate. As the number of batch samples increases, the accuracy tends to decline. However, the accuracy decreases sharply when the times of iterations are 50–60. Thus, the relationship between the number of batch samples and the times of iterations will be balanced when the number of batch samples is 50 in this study. According to the relationship between the times of iterations and accuracy, the two are positively correlated when the times of iterations are less than 10. Nevertheless, the accuracy does not change when the times of iterations are more than 10. It is necessary to ensure the accuracy and calculation performance of the model, and so the times of iterations in this study should be set to 10.

**FIGURE 4:**
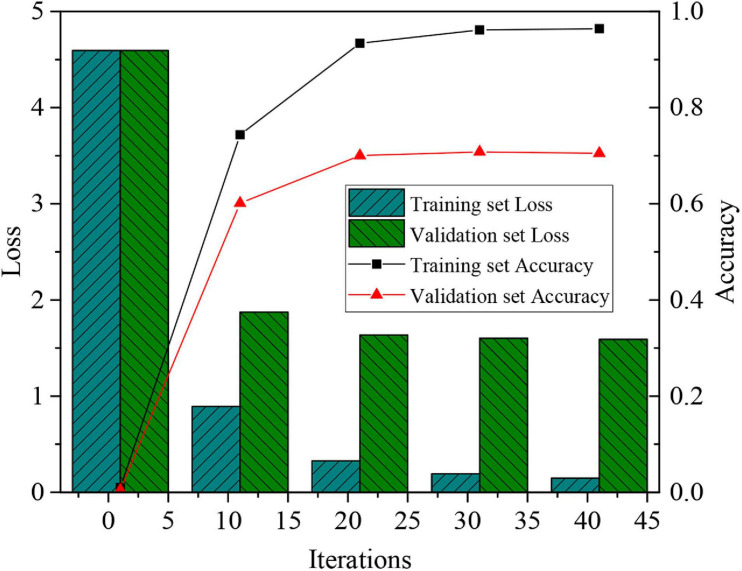
Accuracy and loss value of recognition results based on the gray-scale features.

The video frames with single-channel gray-scale features are trained and verified, and the times of iterations are taken as an independent variable to test the training and verification sets, respectively. Figure 4 shows the experimental results converge when the times of iterations are 20. In addition, the accuracy of the final validation set is stable at 0.71 and the loss value is 1.5.

**FIGURE 5:**
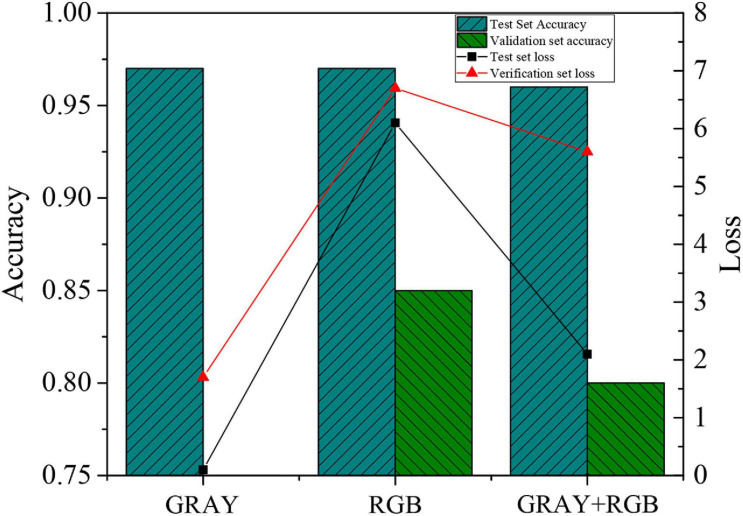
Experimental analysis results of single feature and combined features.

Through analysis of the experimental results of single feature and combined features, the gray-scale feature and RGB feature are combined to detect the accuracy of the model, and the results are presented in Figure 5.

According to Figure 5, the recognition accuracy of the obtained human actions is relatively low when only a gray-scale feature image is used as data input. On the other hand, the combination of gray-scale feature image and RGB image can effectively improve the model recognition accuracy compared with the gray-scale feature image. However, its accuracy is less than that of the RGB image. The loss value of the model drops from 6.7 (RGB image) to 5.6, suggesting that the combination of gray-scale image and RGB image is effective.

**FIGURE 6:**
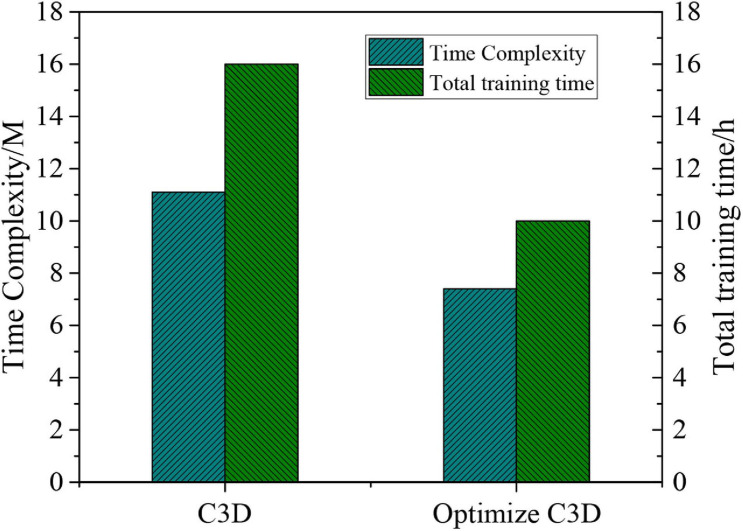
Comparison of time complexity between C3D and optimised C3D.

Finally, the time complexity of C3D and optimised C3D is calculated. The relevant data results are shown in Figure 6, showing that there is a reduction in the time complexity of the optimised model. In addition, the total training time is reduced by 33%.

To sum up, the number of batch samples and times of iteration of the neural network is determined by the gray-scale feature images, and the accuracy and loss values are obtained based on the gray-scale feature recognition results. It is found that the results converge when the times of iterations are 20. Then, the recognition results of single feature and combined features in gray-scale and RGB images are detected, suggesting that the combination of gray-scale and RBG images can reduce image loss and improve the recognition accuracy. Besides, the time complexity of optimised C3D is reduced by 33% compared with C3D. Therefore, the proposed optimised C3D is effective in dealing with the HAR.

## Conclusion

The next action of one athlete can be predicted by recognising the actions in order to explore the psychological changes of fake actions in sports. The optimised C3D is designed based on CNN and the recorded videos of basketball players’ offensive and defensive processes are analysed so that the next actions of players can be predicted in combination with relevant sports psychology theories. The experimental results show that both the number of batch samples and the times of iterations of the network will affect the recognition accuracy of the model. The recognition rate of the optimised C3D is 80% and the loss value is 5.6. However, there are still some deficiencies in this study, including the low overall recognition accuracy of the model and the poor prediction effect of the next actions of athletes. Therefore, continuous improvement is needed in the follow-up research.

## Data Availability Statement

The raw data supporting the conclusions of this article will be made available by the authors, without undue reservation.

## Ethics Statement

The studies involving human participants were reviewed and approved by Beijing Sport University Ethics Committee. The patients/participants provided their written informed consent to participate in this study. Written informed consent was obtained from the individual(s) for the publication of any potentially identifiable images or data included in this article.

## Author Contributions

The author confirms being the sole contributor of this work and has approved it for publication.

## Conflict of Interest

The author declares that the research was conducted in the absence of any commercial or financial relationships that could be construed as a potential conflict of interest.
